# Early Intervention With Cecal Fermentation Broth Regulates the Colonization and Development of Gut Microbiota in Broiler Chickens

**DOI:** 10.3389/fmicb.2019.01422

**Published:** 2019-06-25

**Authors:** Yujie Gong, Hua Yang, Xin Wang, Wenrui Xia, Wentao Lv, Yingping Xiao, Xiaoting Zou

**Affiliations:** ^1^Key Laboratory of Animal Nutrition and Feed Science in East China, Ministry of Agriculture, College of Animal Science, Zhejiang University, Hangzhou, China; ^2^Institute of Quality and Standard for Agro-products, Zhejiang Academy of Agricultural Sciences, Hangzhou, China; ^3^Institute of Plant Protection and Microbiology, Zhejiang Academy of Agricultural Sciences, Hangzhou, China

**Keywords:** fermentation broth, early intervention, gut microbiota, SCFAs, broiler chickens

## Abstract

The aim of this study was to investigate the effect of fermentation broth from broiler cecal content on the colonization and development of the gut microbiota in newly hatched broiler chicks. The fermentation broth was made by a chemostat system using the cecal content from a donor chicken as the source of inoculum. A total of 120 newly hatched broiler chicks were randomly divided into two groups. One group (F group) was orally inoculated with the fermentation broth, and the other (C group) was treated with an equal amount of sterile PBS solution. 16S rRNA gene sequencing was used to investigate the differences in the cecal microbiota of the broiler chickens between the two groups on days 1, 3, 7, 14, and 28. Moreover, the concentrations of short-chain fatty acids (SCFAs) in the cecal contents were analyzed by gas chromatography. The results showed that the abundances of genera *Escherichia–Shigella* and *Enterococcus* decreased sharply in the F group on days 1 and 3 by the early intervention with cecal fermentation broth. In contrast, the relative abundance of the genus *Bacteroides* on days 1, 3, and 7, and the family Ruminococcaceae on days 1, 3, and 28 increased in the F group, respectively. In terms of SCFAs, the concentrations of acetate on day 28, propionic acid on days 1, 3, 7, 14, and 28, butyrate on day 1, and isovalerate on day 14 were significantly higher in the F group compared with the C group. Overall, these results suggest that early intervention with cecal fermentation broth could have beneficial effects on broilers gut health, which might be attributed to the alterations in the gut microbial composition and the increased concentrations of SCFAs.

## Introduction

The gastrointestinal tract of poultry is densely populated with microorganisms, which are considered to have vitally important influences on host health and growth performance (Wielen et al., 2002; Yeoman et al., 2012). In particular, the first species that colonize the gastrointestinal tract have the largest effect on the establishment of intestinal microbiota and the subsequent health and productivity of broiler chickens, as evidenced by the antibiotics and probiotics used to promote growth in the poultry industry (Zhou et al., 2012; Stanley et al., 2014). Broiler chickens represent a specific case for studies focused on host-microbiota interactions. In recent years, some studies have reported that microorganisms can be acquired in the prehatching phase, which come directly from the mother in the oviduct of the hen (Gantois et al., 2010) or from the environment through the pores in the eggshell (Roto et al., 2016). However, among modern production animals, broiler chickens are different in that their parents are not involved in the task of incubating or rearing their young. This separation markedly reduces parental influence on the development of the microbiota. In addition, the implementation of strict hygiene measures by commercial hatcheries reduces the spread of bacteria in the hatching environment to the embryos and newly hatched chicks (Donaldson et al., 2017). Therefore, it is well recognized that the gut microbial community of newly hatched chickens is characterized by low diversity with high instability and is susceptible to modification by exogenous factors such as the intestinal environment (Hooper et al., 2000; Medvecky et al., 2018). Accordingly, the initial developmental period after hatching is a critical stage that leaves a very small window for permanent microbiota remodeling (Fuller, 2010; Baldwin et al., 2018).

The initial inoculation and colonization of the gut microbiota can have an enormous impact on the growth performance and health of broiler chickens (Guarner and Malagelada, 2003; Sears, 2005) as well as on flock microbial uniformity and reproducibility (Stanley et al., 2013). Since the ban on antibiotic growth promoters in many countries because of the increasing occurrence of antibiotic resistance to human pathogens, large-scale chicken farms have experienced challenges regarding prophylaxis and growth promotion (Dibner and Richards, 2005; Huang et al., 2018). Because of the increasing global consumption of chicken, it has become imperative to look for other nutritional strategies to regulate the gut microbiota to improve the growth performance of chickens. One such strategy is to provide newly hatched chickens with probiotics. For instance, Baldwin et al. (2018) inoculated broiler chickens immediately post-hatch with three species of *Lactobacillus*, and as a result they found a tendency for beneficial taxa to be increased and some pathogenic taxa to be reduced in the probiotic-administered group. Furthermore, fecal microbiota transplantation (FMT), which refers to the transfer of the entire fecal microbiota from a healthy donor to a recipient, can also be regarded as a good intervention method. Previous studies have shown that FMT applications can modulate the intestinal microbial community and improve intestinal physiological function (Varmuzova et al., 2016; Hu et al., 2018).

However, the large-scale application of probiotics and FMT on commercial farms, especially farms for the commercial production of broiler chickens, remains uncommon based on restrictions of farming costs and operating procedures. Therefore, in our experiment, an undefined bacterial culture was made by a chemostat system in which the cecal content of a chicken was used as the source of the inoculum. A large amount of fermentation broth can be produced for animal production when the system is stable. Nevertheless, care must be taken when the composition of bacterial species in a final inoculum is not constant. The intervention capacity of fermentation broth can be affected by the various microbial compositions in the ceca of the selected donor chickens and the settings of the chemostat system. Here, we used broiler chickens as the animal model to investigate the effect of early intervention with cecal fermentation broth on the growth performance, the colonization and development of the gut microbiota, as well as the concentrations of short-chain fatty acids (SCFAs) in broiler cecal contents.

## Materials and Methods

### Ethics Statement

The study was conducted according to the Chinese guidelines for animal welfare and approved by the animal welfare committee of the Animal Science College of Zhejiang University (Hangzhou, China).

### Preparation of the Cecal Fermentation Broth

The preparation of the cecal fermentation broth required a single-stage chemostat system, which was used in our previous study (Yin et al., 2010). In brief, this system was anaerobically maintained by continuous flow of pure nitrogen into the medium reservoir and working vessel. The growth medium was configured by Genovese’s description (Genovese et al., 2003). The temperature was maintained at 37°C and the pH was controlled at 6.2 when the system was operating.

A 180-day-old broiler chicken, with no history of gastrointestinal diseases or record of antibiotic use, was selected from a mountain village in Jinhua, Zhejiang province and used as the cecal contents donor. A 10% suspension was made by homogenizing the fresh cecal contents with sterile phosphate buffered saline (PBS). The chemostat system was stabilized at the designated temperature and pH before the suspension began to flow into the working vessel via a peristaltic pump. Subsequently, the suspension was augmented by anaerobic fermentation in the chemostat system. Based on the steady-state condition of chemostat system, we chose to use the broth after 11 days of fermentation as the inoculum for newly hatched chicks.

### Animals and Sampling

A total of 120 newly hatched broiler chicks were purchased from a local commercial hatchery on the day of hatching and were randomly divided into two groups, which were named the control group (C group) and fermentation broth group (F group). Each group consisted of 8 coops with 8 chickens per coop. The broilers in the F group were orally inoculated with 0.5 mL of fermentation broth on day 0 (within 2 h post-hatching). A volume of 0.5 mL of sterile PBS was given to the broilers of the C group in the same manner. The broilers were kept in an environmentally controlled animal facility. For the 1st week, the environmental temperature was kept at 35°C and then gradually reduced to 28°C by the end of the experiment. During the 1st week, 24 h/day of light were provided, and afterwards reduced to 22 h/day. None of the broiler chickens were administered antibiotics or other drugs throughout the experiment.

On days 1, 3, 7, 14, and 28, eight broilers from each group were weighed individually after 4 h of fasting and then sacrificed under chloroform anesthesia. The cecal contents were collected from 8 broilers per group at each sampling time point. A total of 80 cecal content samples were collected on ice, immediately frozen in liquid nitrogen, and then stored at -80°C until analysis.

### DNA Extraction and Purification

Bacterial DNA was extracted from the cecal content samples using the QIAamp DNA Stool Mini Kit (Mo Bio Laboratories, San Diego, CA, United States) according to the manufacturer’s instructions. The quantity and quality of DNA extracted from these samples were determined by a Nanodrop 2000 spectrophotometer (Thermo Fisher Scientific, Wilmington, DE, United States). The V4–V5 hypervariable region of the 16S rRNA gene was then amplified by polymerase chain reaction (PCR) using two universal eubacterial primer pairs 515F (5′-GTGCCAGCMGCCGCGG-3′) and 907R (5′-CCGTCAATTCMTTTRAGTTT-3′), as described by Xiong et al. (2012). All primers were synthesized by Invitrogen Life Technologies (Shanghai, China). The PCR amplification was conducted using TransGen AP221-02: TransStart FastPfu DNA polymerase (TransGen Biotech, Beijing, China) and performed in a GeneAmp 9700 thermal cycler (Applied Biosystems, Foster City, CA, United States). The PCR reaction conditions were as follows: 95°C for 3 min followed by 27 cycles of 95°C for 30 s, 55°C for 30 s, and 72°C for 45 s, with a final extension at 72°C for 10 min. PCR reactions were performed in triplicate with each 20 μL of reaction mixture, containing 4 μL of 5 × FastPfu buffer, 2 μL of 2.5 mM dNTPs, 0.8 μL of each primer (5 μM), 0.2 μL BSA, 10 ng of template DNA, and H_2_O to a final volume of 20 μL. The PCR products were excised from a 2% agarose gel after electrophoresis, and purified using the AxyPrep DNA Gel Extraction Kit (AXYGEN, Union City, CA, United States) and then quantified using QuantiFluor-ST (Promega, Madison, WI, United States).

### 16S rRNA Gene Sequencing and Data Processing

The V4–V5 region of the 16S rRNA gene was sequenced on the Illumina MiSeq PE250 sequencing platform according to the manufacturers’ suggested protocols. The 16S rRNA-derived sequence inventories were processed using the Quantitative Insights into Microbial Ecology (QIIME) (Caporaso et al., 2010). The Illumina raw data was filtered to remove low quality reads. The sequences with a mean quality score of no less than 20 and a length longer than 250 bp were retained. The UPARSE software (version 7.1^1^) was used for read clustering and the cutoff (based on 97% similar identity) for operational taxonomic Units (OTUs). In addition, the UCHIME method was used to remove chimeric OTUs from further analysis (Edgar, 2010). Finally, the taxonomy assignment was performed with the Ribosomal Database Project (RDP, Release 11.1^2^) classifier Bayesian algorithm to analyze the clustered OTUs against the 16S reference database Silva (Release 119^3^).

Alpha diversity (Chao1 and Shannon index) was assessed using Mothur version 1.22.2 (Schloss et al., 2009). Beta diversity was calculated based on unweighted UniFrac distances by QIIME. An unweighted UniFrac PCoA based on the OTUs was performed to provide an overview of the differences in microbial diversity and composition in the cecal contents of broiler chickens between the two groups. A LefSe analysis was performed to identify which microbes that significantly influenced the difference between samples (Segata et al., 2011). The more intuitive heatmap analysis was used to indicate the similarities and differences in the community composition of the samples.

### Analysis of SCFAs in the Cecal Contents by Gas Chromatography

Four samples from each group at each sampling time point were used to determine the concentrations of short chain fatty acids (SCFAs), including acetate, propionate, butyrate, valerate, isobutyrate, and isovalerate, according to the previously described method by Wang et al. (2017), with slight modifications. In brief, 100 mg of cecal content was homogenized with 1 mL of sterile PBS and centrifuged for 10 min at 12,000 rpm and 4°C. Then, a 500 μL aliquot of the supernatant fluid was diluted with 100 μL of 25% (w/v) metaphosphoric acid solution. The mixture was incubated for 24 h at -20°C and then centrifuged for 10 min at 12,000 rpm and 4°C. Finally, the collected supernatant was filtered through a 0.22 μm syringe filter and injected into a Shimadzu GC-2010 ATF instrument for the determination of SCFAs. The carrier gas was N_2_ (pressure, 12.5 Mpa and flow, 18 mL min^-1^), the temperature of the injector and detector was 180°C, and the column was gradually heated from 80 to 170°C at a rate of 4°C min^-1^.

### Statistical Analysis

The experimental data, including weight, the relative abundance of bacteria, and the concentrations of SCFAs, were analyzed with SPSS 22.0 (IBM, New York, NY, United States). The differences between the two groups were examined for significance by an independent-sample *t*-test to conduct the variance analysis. The results presented in this article are shown as the mean ± SEM and were considered significant at *P* < 0.05.

## Results

### Microbial Composition of the Final Inoculum

**FIGURE 1:**
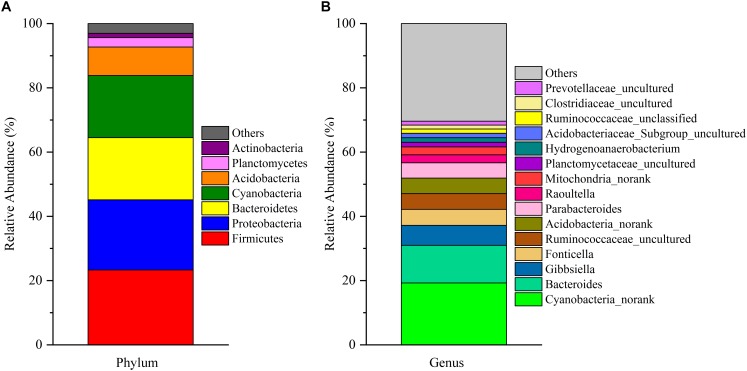
The composition of the microbial community in the fermentation broth. Panel **(A)** shows the community composition of the microbiota at the phylum level. Panel **(B)** displays the community composition of the microbiota at the genus level.

After removing incorrect and chimeric sequences, we obtained 54,427 high-quality bacterial V4–V5 16S rRNA sequence reads from the cecal fermentation broth. Almost all sequences were between 350 and 400 bp, with an average read length of 396.21 bp. A total of 1,466 OTUs were identified at a sequence similarity level of 97% and then assigned to 25 phyla, 64 classes, 137 orders, 250 families and 465 genera. Seven dominant phyla with a relative abundance > 1% are shown in Figure 1A. There were almost no differences in the relative abundances of Firmicutes, Proteobacteria, Bacteroidetes, and Cyanobacteria, each of which accounted for approximately 20% and together accounted for greater than 80% of the entire group of OTUs. A genus-level microbiota analysis revealed that the microbiota in the fermentation broth was dominated by *Cyanobacteria_norank* and *Bacteroides* (Figure 1B), which belong to the Cyanobacteria and Bacteroidetes phyla, respectively. In addition, the microbial complexity in the fermentation broth was estimated on the basis of alpha-diversity indexes. Chao1 (1,636) and ACE (1,663) indexes were used to estimate species richness, while Shannon’s index (4.40) and Simpson index (0.59153) were used to indicate species diversity (Supplementary Table S1).

### Growth Performance of the Boiler Chickens

**FIGURE 2:**
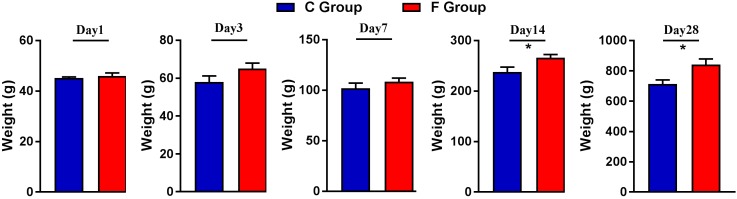
Weights of the broiler chickens on days 1, 3, 7, 14, and 28. *n* = 6 per group. C group, control group; F group, fermentation broth group.

The effect of early intervention with cecal fermentation broth on the growth performance of the broiler chickens is presented in Figure 2. Compared with the C group, the broilers of the F group grew faster throughout the entire experimental period and showed significantly higher body weight on days 14 and 28 (*P* < 0.05).

### Early Intervention Alters the Diversity and Structure of the Broiler Cecal Microbiota

Cecal content samples from 80 chickens were obtained to assess the microbiota composition by MiSeq-mediated sequencing. In this experiment, we randomly subsampled all the samples to 31,570 high-quality sequencing reads to avoid bias caused by different sequencing depths. In total, 6,195 OTUs (97% sequence similarity within an OUT) were obtained from all samples. Rarefaction curves (Supplementary Figure S1) were created according to the number of OTUs calculated for the average subsample of the sequenced read pools in each group. The number of OTUs increased sharply before the average rarefaction curve tended to reach a plateau, which indicated that the sequencing data was deemed adequate to cover the vast majority of biodiversity contained within the samples according to the number of OTUs, as the rarefaction curves tended toward saturation.

**FIGURE 3:**
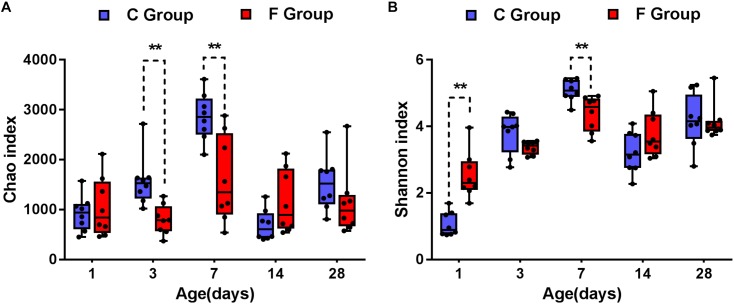
Alpha diversity of the gut bacterial community of the broiler chickens in the two groups at each sampling time point. Panel **(A)** indicates the species richness of the bacteria. Panel **(B)** shows the community diversity of the bacteria. Asterisks indicate statistically significant differences between the two groups: ^∗^*P* < 0.05, ^∗∗^*P* < 0.01. *n* = 8 per group. C group, control group; F group, fermentation broth group.

The data corresponding to the differences in the richness and diversity of the cecal microbiota between the C and F groups are shown in Figure 3. Early intervention with cecal fermentation broth decreased the richness of gut microbiota on days 3 and 7, as evidenced by the observed OTUs (Figure 3A). The alpha diversity estimated by Shannon’s index was strongly affected by early intervention with cecal fermentation broth, especially in the early stages. Interestingly, there was a turning point between days 1 and 7. Shannon’s index significantly increased in the F group compared with that in the C group on day 1 (*P* < 0.01); however, opposite results appeared on day 7 (Figure 3B).

**FIGURE 4:**
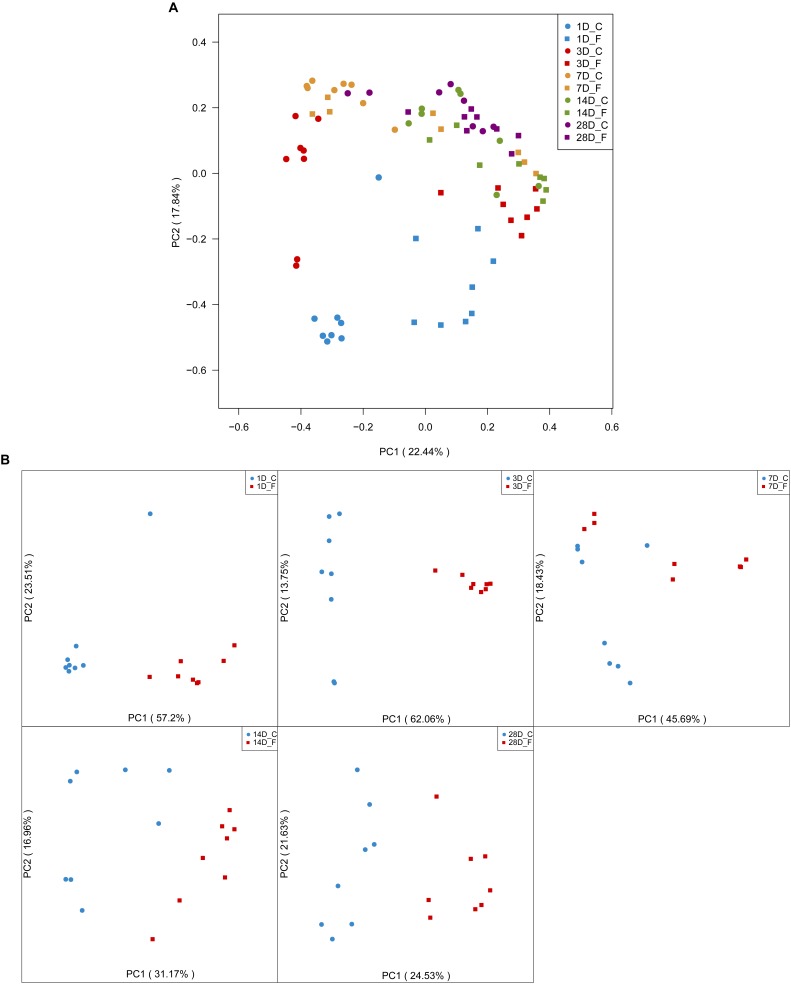
Evaluation of the beta diversity of the broiler chickens in the two groups. Panel **(A)** depicts the overall characteristics of all 80 samples by a principal coordinate analysis. Panel **(B)** illustrates the differences in gut microbial community structure between the two groups at each sampling time point. *n* = 8 per group. C group, control group; F group, fermentation broth group.

Additionally, the Bray–Curtis similarity metric was used to evaluate the beta diversity across the samples (namely, diversity among individuals). As shown in Figure 4A, all samples were distributed into ten different clusters based on groups and ages. The analysis of day-age on the PCoA plot displayed a heterogeneous distribution of the samples. The gut microbiota of 1-day-old broilers were obviously different from other age samples, whereas samples on days 14 and 28 exhibited much higher similarity. On the other hand, the PCoA analysis in considering the effect of early intervention revealed that the bacterial community structures of the C group and the F group were distinctly separated at each sampling time (days 1, 3, 7, 14, and 28) (Figure 4B).

### Effects of Early Intervention on Cecal Microbiota Composition

**FIGURE 5:**
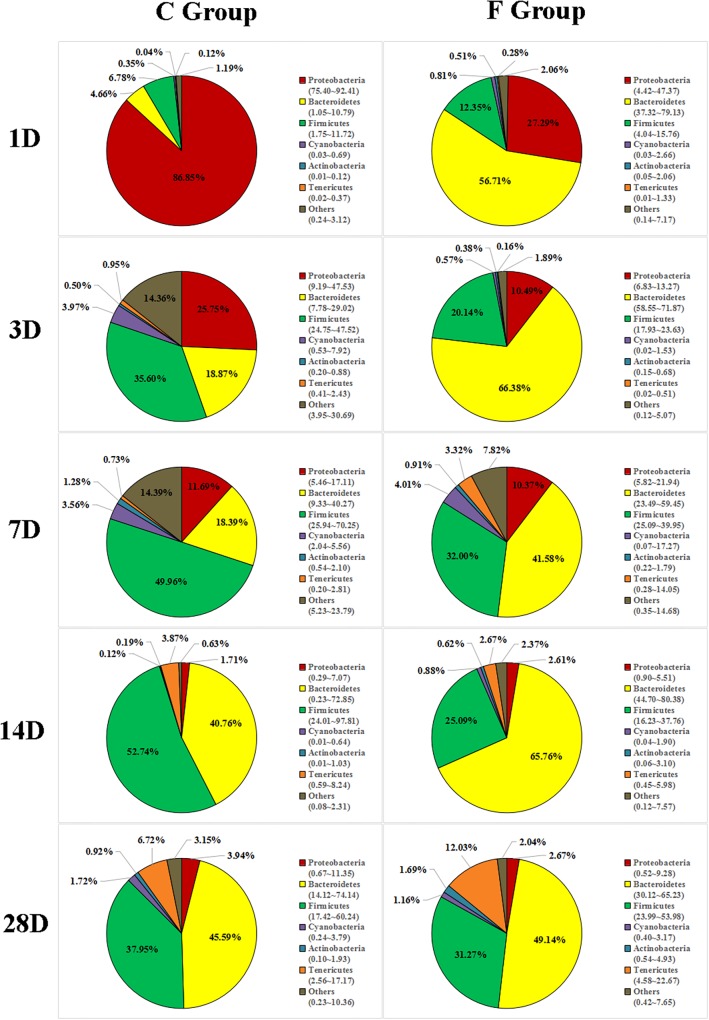
Phylum-level community composition of the cecal microbiota between the C group and the F group. Only the 6 dominant phyla of each group are shown at each sampling time point. *n* = 8 per group. C group, control group; F group, fermentation broth group.

**FIGURE 6:**
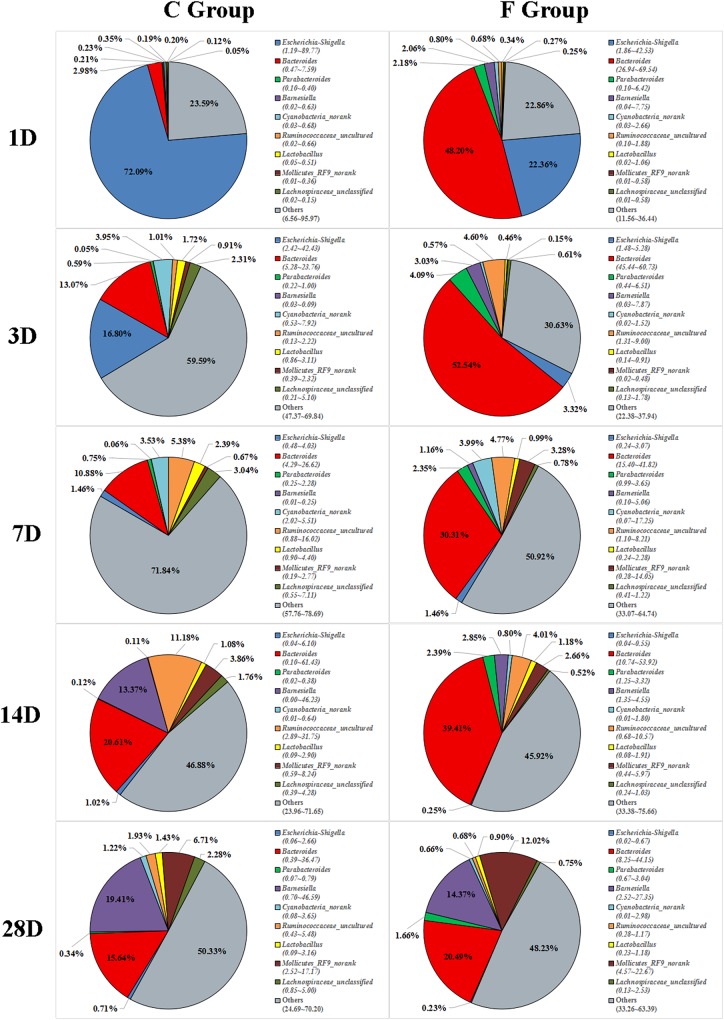
Genus-level community composition of the cecal microbiota between the C group and the F group. Only the 9 dominant genera in each group are shown at each sampling time point. *n* = 8 per group. C group, control group; F group, fermentation broth group.

The overall cecal microbiota composition of broiler chickens significantly varied between the C and F groups and was associated with the early intervention with cecal fermentation broth. The relative abundances of the 6 most abundant phyla (Proteobacteria, Bacteroidetes, Firmicutes, Cyanobacteria, Actinobacteria and Tenericutes) across the two groups are shown in Figure 5. The inspection of the predicted taxonomic profiles at the phylum level for all samples revealed that the phylum Bacteroidetes, with a mean relative abundance ranging from 41.58 ∼ 66.38%, was the most abundant phylum in the cecal microbiota community of the intervened chickens at all age points. Firmicutes was the second dominant phylum on days 3 (20.14%), 7 (32.00%), 14 (25.09%), and 28 (31.27%). While the relative abundance of Proteobacteria (27.29%) was ranked second on day 1, followed by Firmicutes (12.35%). A higher proportion of the phylum Bacteroidetes was observed in the F group compared to that in the C group throughout the experiment. In contrast, we found that a dramatic decrease in the abundance of Proteobacteria was observed on days 1 (27.29% vs. 86.85%) and 3 (10.49% vs. 25.75%) in the F group. More importantly, the abundance of Proteobacteria displayed a sustained downward trend with age both in the C and F groups, which was shown by the smaller proportion of Proteobacteria on day 28 than on day 1. At the genus level (Figure 6), the bacterial taxa were quite different at all age points between the C group and the F group. *Bacteroides* was the most abundant genus identified in the F group at all age points, with the highest value on day 3 (52.54%) and the lowest value on day 28 (20.49%). *Escherichia–Shigella* was the most predominant genus, with an average abundance of 72.09% in the control chickens on day 1, but the level decreased with increasing age. In contrast, *Barnesiella* and *Mollicutes_RF9_norank* had a lower relative abundance in the early stage of the experiment but were predominant on day 28 in both groups.

**FIGURE 7:**
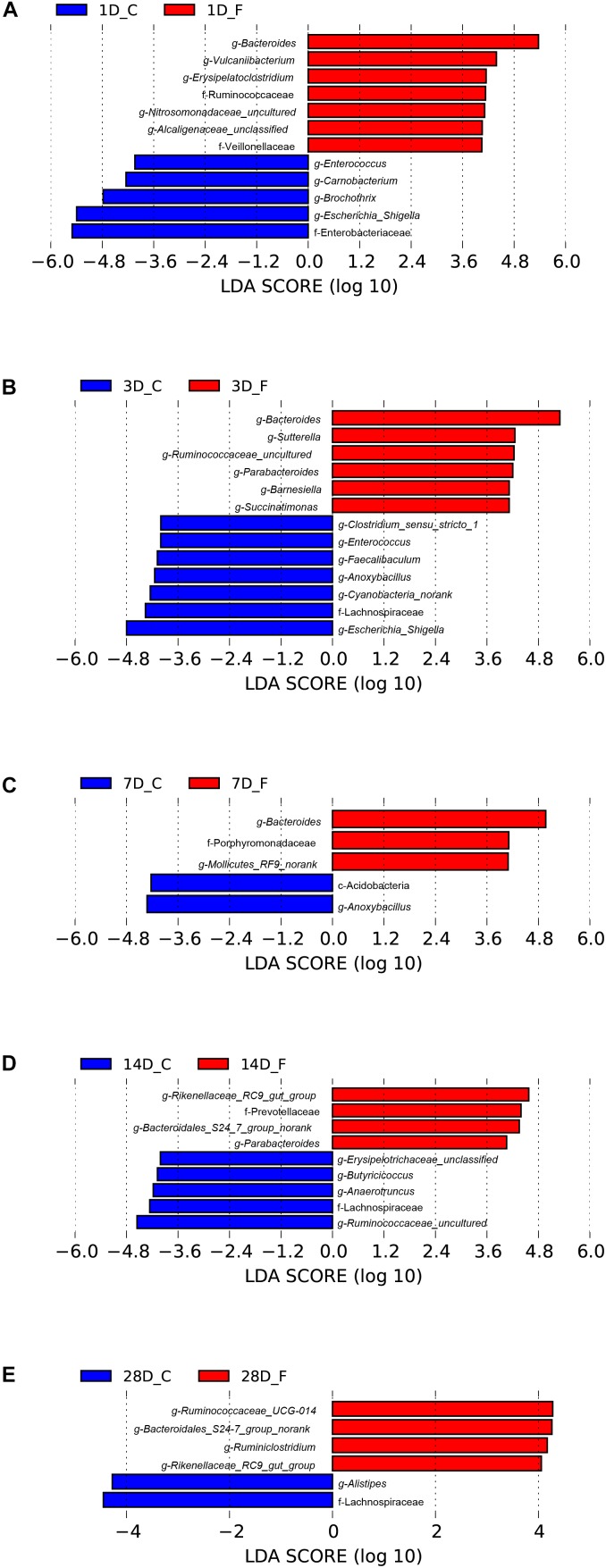
Differentially abundant bacteria between the C group and the F group at each sampling time point. Histograms of linear discriminant analysis (LDA) scores (threshold ≥ 4) on days 1 **(A)**, 3 **(B)**, 7 **(C)**, 14 **(D)**, and 28 **(E)** are plotted. *n* = 8 per group. C group, control group; F group, fermentation broth group.

Significant differences in the relative abundance of bacteria in the cecal microbiota between the C group and the F group at a certain age were further identified using a LefSe analysis, which not only emphasizes statistical significance but also biological consistency (Segata et al., 2011). Based on the logarithmic LDA score of 4.0 as the cutoff, we found that 12 taxa on day 1 were significantly affected by early intervention, followed by 13 taxa on day 3, 5 taxa on day 7, 9 taxa on day 14, and 6 taxa on day 3 (Figure 7). Among the significantly different taxa, *Bacteroides* was the most abundant bacteria in the intervened chickens at the genus level from days 1 to 7. Furthermore, early intervention significantly increased the abundance of bacteria belonging to the family Ruminococcaceae on days 1, 3, 28 and the relative abundance of the order Bacteroidales (including the genera *Bacteroidales_S24-7_group_norank* and *Rikenellaceae_RC9_gut_group*) on days 14 and 28. However, the relative abundance of the genus *Escherichia–Shigella* was significantly lower in the intervened chickens on days 1 and 3. To comprehensively compare the relative abundance of the bacteria across samples, the genera with the top 50 relative abundances at each sampling time point were shown in the heatmap to determine the similarities and differences in the community composition of the samples (Supplementary Figure S2). An increasing trend of the genus *Bacteroides* and a sharp downward trend of the genus *Escherichia–Shigella* were observed in the F group during early life.

### Core Microbial Genera in the Cecal Contents of the Broiler Chickens

**FIGURE 8:**
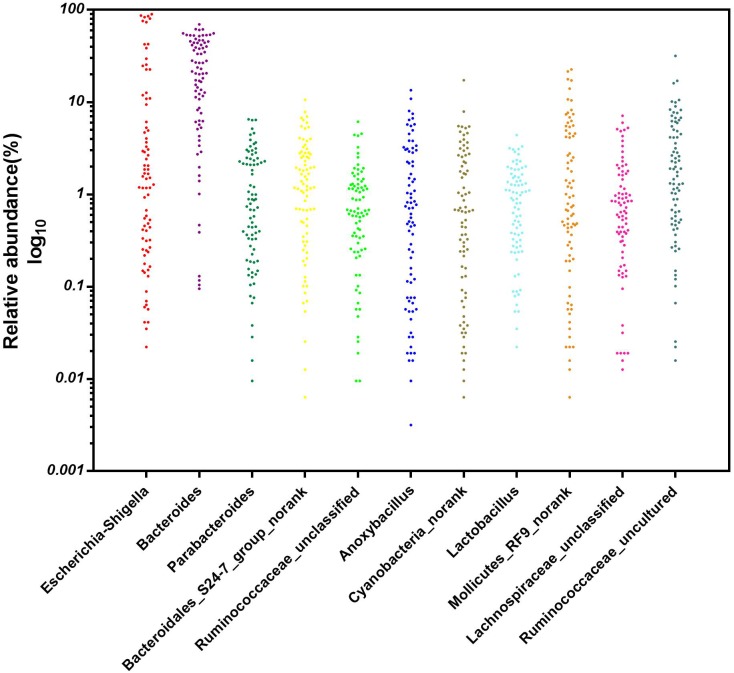
The core microbial genera in the cecal content of the broiler chickens. *n* = 80.

A particular part of our study was to investigate whether core microbial genera were shared among all 80 cecal samples (8 repetitions per group on days 1, 3, 7, 14, and 28) and could be considered the basic genera for studying the cecal microbiota of broiler chickens. To address this question, the genera with the top 35 relative abundances at each sampling time point were chosen as the reference database. We found 11 predominant genera in all sampled individuals (*n* = 80) (Figure 8). These 11 core genera were distributed among five phyla. Five out of the 11 genera were from the phylum Firmicutes (the genera *Ruminococcaceae_unclassified*, *Anoxybacillus*, *Lactobacillus*, *Lachnospiraceae_unclassified*, and *Ruminococcaceae_uncultured*), and three from the phylum Bacteroidetes (the genera *Bacteroides*, *Parabacteroides*, and *Bacteroidales_S24-7_group_norank*), with the remaining being in the phyla Proteobacteria (the genus *Escherichia–Shigella*), Cyanobacteria (the genus *Cyanobacteria_norank*) and Tenericutes (the genus *Mollicutes_RF9_norank*), respectively. The relative abundance of these genera varied greatly by ages, these 11 genera were consistently detected in the cecal contents of the broiler chickens from days 1 to 28, suggesting that early intervention with cecal fermentation broth modified the relative abundances but not the presence or absence of these specific microbial genera.

### Concentrations of SCFAs in the Cecal Contents of the Broiler Chickens

**FIGURE 9:**
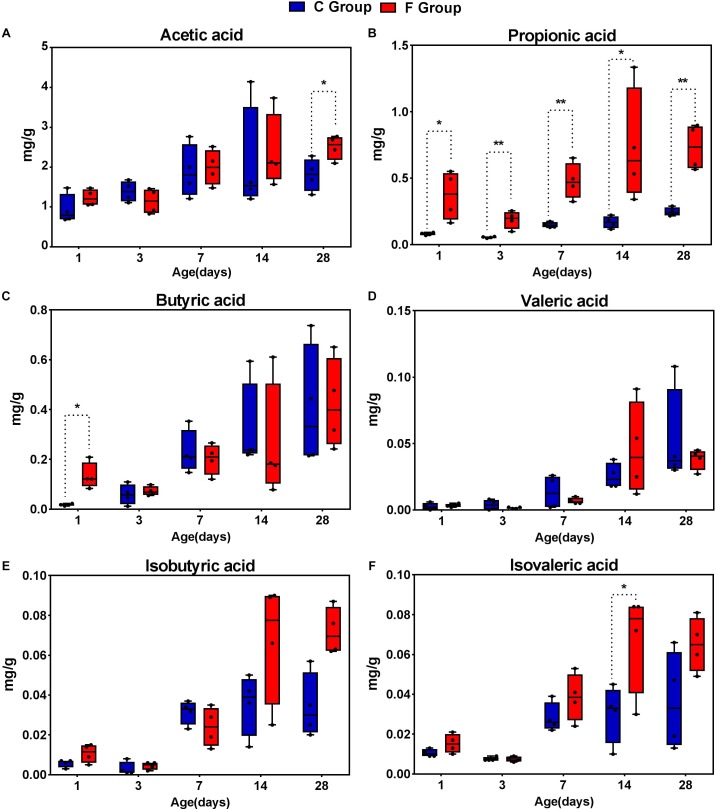
The concentrations of short-chain fatty acid (SCFAs) in the cecal contents of the broiler chickens between the C group and F group. *n* = 4 per group. C group, control group; F group, fermentation broth group. **(A)** Acetic acid, **(B)** Propionic acid, **(C)** Butyric acid, **(D)** Valeric acid, **(E)** Isobutyric acid, and **(F)** Isovaleric acid.

In order to describe whether the observed gut microbial changes resulting from early intervention affected gut function, the concentrations of SCFAs were determined (Figure 9). The acetate concentration in the F group, accounting for the largest proportion of total SCFAs, was significantly higher (*P* < 0.05) than that in the C group on day 28. Likewise, elevated concentrations of butyrate and isovalerate were observed in the intervened chickens on days 1 and 14, respectively (*P* < 0.05). In addition, our results suggest that early intervention markedly increased the concentration of propionic acid at all sampling time points, particularly achieving extremely significant statistical significance (*P* < 0.01) on days 3, 7, and 28. In contrast, no differences in the production of isobutyrate and valerate were observed in the cecal contents between the C group and the F group. Taken together, these results indicate that the early intervention with cecal fermentation broth significantly enhanced the concentrations of SCFAs, especially propionic acid.

## Discussion

This investigation aimed to determine whether early intervention with cecal fermentation broth, produced through the use of a chemostat system, could have a beneficial effect on the colonization and development of the gut microbiota in newly hatched chicks. To our knowledge, this is a new strategy to regulate the colonization and development of the gut microbiota. In the present study, an average of 31,570 high quality sequence readings was obtained per cecal content sample, which was much higher than that of previously published studies on chicken gut microbiota (Pourabedin et al., 2015; Vermeulen et al., 2017). Therefore, this high number of sequences enabled us to provide a more comprehensive analysis of species richness and cecal microbiota diversity. The highest levels of richness and diversity of the cecal microbiota in the broiler chickens were detected on day 7. Unexpectedly, our results showed that species richness of gut microbiota was significantly higher in the C group on days 3 and 7 when compared to the F group. We speculate that this result may be attributed to some enriched pathogenic bacteria in the C group. In addition, we observed that the Shannon index of gut microbiota in the intervened chickens was significantly increased following early intervention on day 1, while the opposite trend appeared on day 7, suggesting that early intervention could exert inconsistent effects on α-diversity of gut microbiota in broilers at different ages. Generally, high α-diversity of gut microbiota was favorable for the overall health and productivity of livestock animals (Zhang et al., 2016). In contrast, a new study revealed that the concept for understanding diversity in host-associated microbial communities is not as simple as “more diversity is better” (Reese et al., 2018). Sometimes limited diversity is desirable since not all microbes are beneficial, whether because they are pathogens or because they are just cheating strains providing little function to the host (Foster et al., 2017). Besides, the microbial community structure (β-diversity) between the two groups at all sampling time points was compared using PCoA of the unweighted UniFrac distance. These PCoA plots showed that microbial communities from the F group separated clearly from those of the C group at each sampling time point. The overall sample distribution showed that the gut microbiota of newly hatched chicks (day 1) were highly variable and obviously different from other samples (days 3, 7, 14, and 28), reflecting the initiation of gut microbial communities is an optimal period for modulating gut microbiota.

The most complex microbial community within the chicken gut is the one that resides in the cecum (Rinttilä and Apajalahti, 2013). In this study, the dominant phyla detected in the cecal contents were the phyla Bacteroidetes, Firmicutes, and Proteobacteria, which was in accordance with the results of the dominant phyla in the cecal fermentation broth. Moreover, the same results have been reported in other studies (Sofka et al., 2015; Mancabelli et al., 2016). Nevertheless, the composition of gut microbiota might also be influenced by age. From days 14 to 28, Tenericutes gradually became one of the main bacterial phyla in both C and F groups. Similarly, an investigation of the cecal microbiomes of the broilers by Sakaridis et al. (2018) suggested that Tenericutes was indeed one of the dominant phyla of bacteria at a certain age. Additionally, we found that early intervention could change the relative abundances of dominant phyla, as shown by a significantly higher proportion of Bacteroidetes and a remarkable drop in the proportion of Proteobacteria in the F group compared to the C group. The phylum Bacteroidetes has been associated with short chain acid metabolism, especially for the synthesis of propionate (Pandit et al., 2018). The decrease in the relative abundance of the phylum Proteobacteria may indicate that the broilers in the F group have a healthier intestinal environment, as the phylum Proteobacteria includes a wide variety of pathogenic bacteria (Dai et al., 2018). At the genus level, the most meaningful result for early intervention was the significant reduction in the presence of *Escherichia–Shigella* on days 1 and 3. The genus *Escherichia–Shigella*, which is composed of many *E. coli* strains such as *E. coli* O157:H7, is considered to be a genus that includes opportunistic pathogenic bacteria. Previous studies have documented that the genus *Escherichia–Shigella* can destroy the intestinal structure and have proinflammatory activities through multiple pathways, such as the production of virulence factors (Kaur and Ganguly, 2003), resulting in an increased risk of infection and diarrhea in the host (Sousa et al., 2010). Fortunately, the relative abundance of *Escherichia–Shigella* in the cecal contents of broiler chickens was found to be negatively correlated with age. Beginning at day 7, the proportion of *Escherichia–Shigella* in both groups was significantly reduced to a relative abundance of less than 1% at day 28. These results suggest that potential pathogens might mainly be present in newly hatched chicks, which is the main factor for the chicks’ susceptibility to disease. Additionally, another difference in the cecal microbiota composition at the genus level was detected in the F group compared to the C group which consisted of a continuous increase in the relative abundance of *Bacteroides* (48.20% vs. 2.98%, 52.54% vs. 13.07%, 30.31% vs. 10.88%, 39.41% vs. 20.61%, and 20.49% vs. 15.64% on days 1, 3, 7, 14, and 28, respectively). The benefits of *Bacteroides* are well known. They are effective degraders of indigestible carbohydrates, including cellulose and starch (Salyers et al., 1977), which may result in the improved growth performance of the intervened chickens.

Excluding the dominant bacteria, a LefSe analysis identified other representative species as biomarkers to distinguish the microbiota of the two groups. For example, the family Ruminococcaceae, with a number of SCFA producers, was significantly enriched in the intervened chickens, and these bacteria are considered to be dominant players in the degradation of diverse polysaccharides and fibers (Hooda et al., 2012). With the maturation of gut microbiota, the relative abundance of *Ruminococcaceae_UCG-014* in the intervened chickens on day 28 was still significantly higher than that in the C group. *Ruminococcaceae_UCG-014* is a common genus reported in the chicken cecum (Mohd et al., 2015), which has been associated with the maintenance of gut health and has the enzymatic capability to degrade cellulose and hemicellulose (Louis and Flint, 2009). In contrast, a relative increase of the genus *Enterococcus* was evident in broilers of the C group. *Enterococcus*, which was formerly regarded as a bacterium with minimal clinical impact, has emerged as an important poultry pathogen (Dolka et al., 2017). Taken together, the results from screening the representative species reflect that early intervention is effective in promoting intestinal health by encouraging the growth of beneficial species and inhibiting the proliferation of pathogenic bacteria. In the current study, we were particularly interested in determining the core microbial genera, which could be regarded as the basic genera in the cecal microbiota of the broiler chickens. Here, 11 genera were detected in all broilers, regardless of group or age, and were identified as the core cecal microbiomes in broilers. In fact, most of these genera were classified as being in the family Ruminococcaceae and the order Bacteroidales, which is in accordance with results on the dominant bacteria. However, these core genera also contained some pathogenic bacteria, suggesting that their pathogenicity mainly depends on the relative abundance of pathogens. Therefore, pathogenicity can be weakened by intervening in the colonization and development of gut microbiota to reduce the relative abundance of pathogenic bacteria.

As a link between gut microbiota and its host, metabolites and nutrients can be provided and supplied by intestinal microbiota through their metabolism (Yadav et al., 2017). As an important source of energy for enterocytes, SCFAs are primarily produced by bacterial fermentation in the gut and are vital for intestinal health (Sunkara et al., 2012). Thus, SCFAs are of particular importance and frequently used to assess bacterial metabolism in the intestine (Awad et al., 2016). In the present study, the six main species of SCFAs were analyzed. The results showed a significant increase in the concentrations of SCFAs in the cecal contents of the intervened chickens. This may be due to the alterations in the microbial composition which resulted from the early intervention with cecal fermentation broth. Among the SCFAs, the concentration of propionate, based on our results, increased significantly at all age points in the intervened chickens. One reason for this result may be the co-occurring increases in the relative abundance of propionate-producing bacteria. As an example, the increasing trend in the presence of genus *Bacteroides* was similar to the increasing trend of propionate in the intervened chickens. Salminen et al. (1998) confirmed such a connection in their research that *Bacteroides* plays a key role in the production of propionate. In return, the propionate plays an important role in the maintenance of gut health and has anti-inflammatory effects (Canani et al., 2011). Furthermore, increased concentrations of SCFAs can increase intestinal acidity, which is associated with pathogen suppression (Rehman et al., 2007). For instance, some studies have revealed that SCFAs such as butyrate and propionate have inhibitory effects on *Salmonella*, which is an important foodborne pathogen that is ubiquitous worldwide and frequently infects poultry flocks (Van Immerseel et al., 2004; Vermeulen et al., 2017). Therefore, we suggest that early intervention with cecal fermentation broth could reduce the colonization of some pathogenic bacteria by the acidic intestinal environment resulting from the increased concentrations of SCFAs in the cecal contents of broiler chickens.

## Conclusion

Early intervention with cecal fermentation broth can modulate the colonization and development of the gut microbiota in broiler chickens. The reduction of pathogenic bacteria, the increase of beneficial bacteria and the increase in the concentration of SCFAs are conducive to broiler intestinal health. Therefore, it may be possible to develop new intervention strategies to induce desirable changes in the gut microbiota to enhance growth and productivity of broiler chickens. This is meaningful for large-scale animal production because these new strategies may have similar or improved benefits and lower costs compared to commercial probiotics.

## Author Contributions

YX, XZ, and HY designed the experiments. YG, WX, and XW conducted the experiments. YG, WX, and WL collected the samples. YX and WL analyzed the data. YG wrote the manuscript. YX, XZ, and HY edited the manuscript. All authors read and approved the final manuscript.

## Conflict of Interest Statement

The authors declare that the research was conducted in the absence of any commercial or financial relationships that could be construed as a potential conflict of interest.

## Footnotes

1 http://drive5.com/uparse/

2 http://rdp.cme.msu.edu/

3 http://www.arb-silva.de
