# Supplementary material for: Early Intervention With Cecal Fermentation Broth Regulates the Colonization and Development of Gut Microbiota in Broiler Chickens
Source: Front Microbiol. 2019 Jun 25;10:1422. doi: 10.3389/fmicb.2019.01422 (PMC6603130; doi:10.3389/fmicb.2019.01422)
Supplement: Supplementary file 1 [file Data_Sheet_1.doc]

**Supplementary Table S1.** Characteristics of the species richness and diversity in the final inoculum.

| Sample ID | ACE | Chao1 | Shannon’s index | Simpson |
| --- | --- | --- | --- | --- |
| FB | 1663 | 1636 | 4.40 | 0.059135 |

FB: fermentation broth.


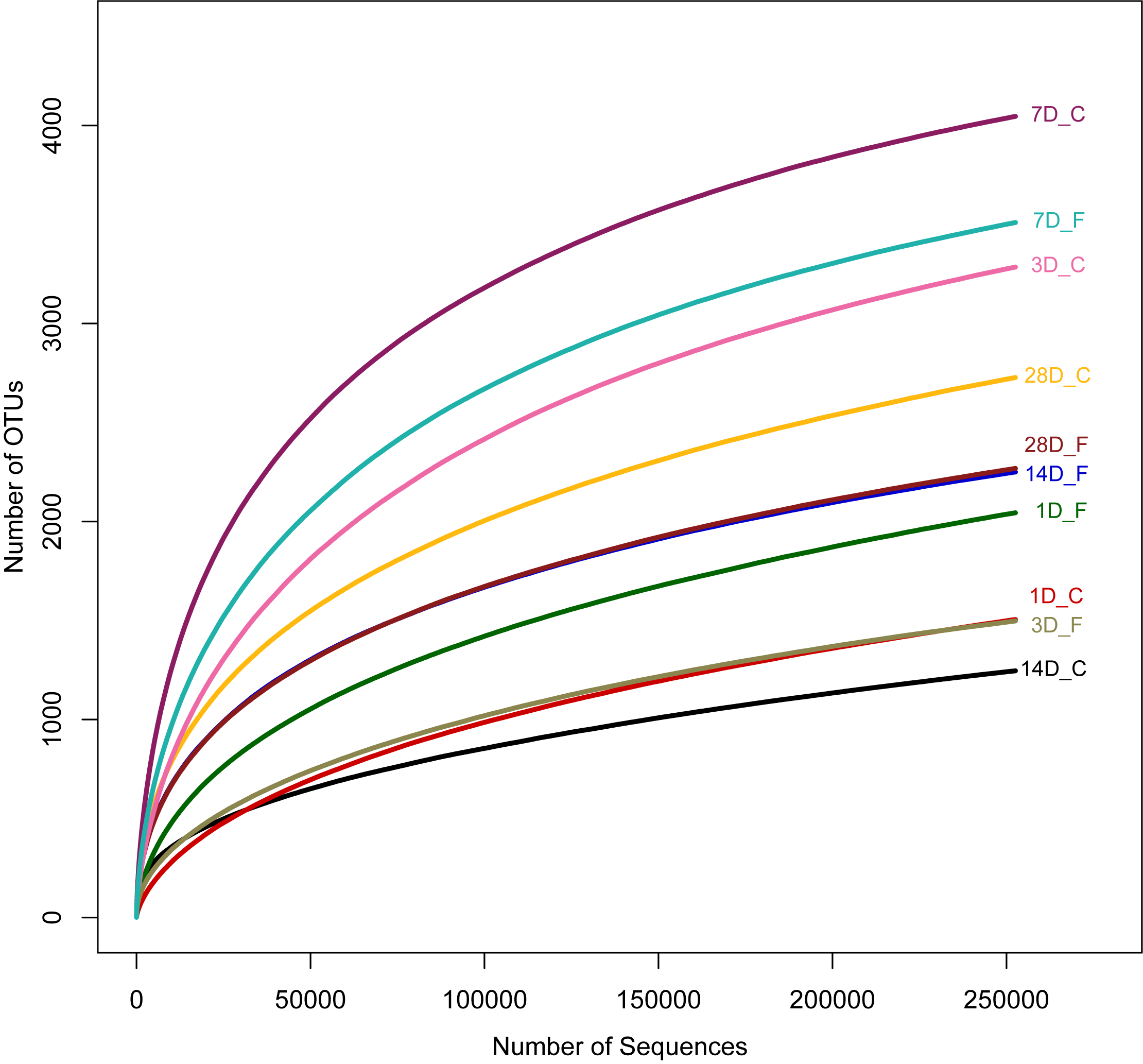
**Supplementary Figure S1.** The average rarefaction curves representing the number of OTUs with the increasing sequencing depth of cecal content samples on days 1, 3, 7, 14 and 28. *n* = 8 per group. C Group, control group; F group, fermentation broth group.


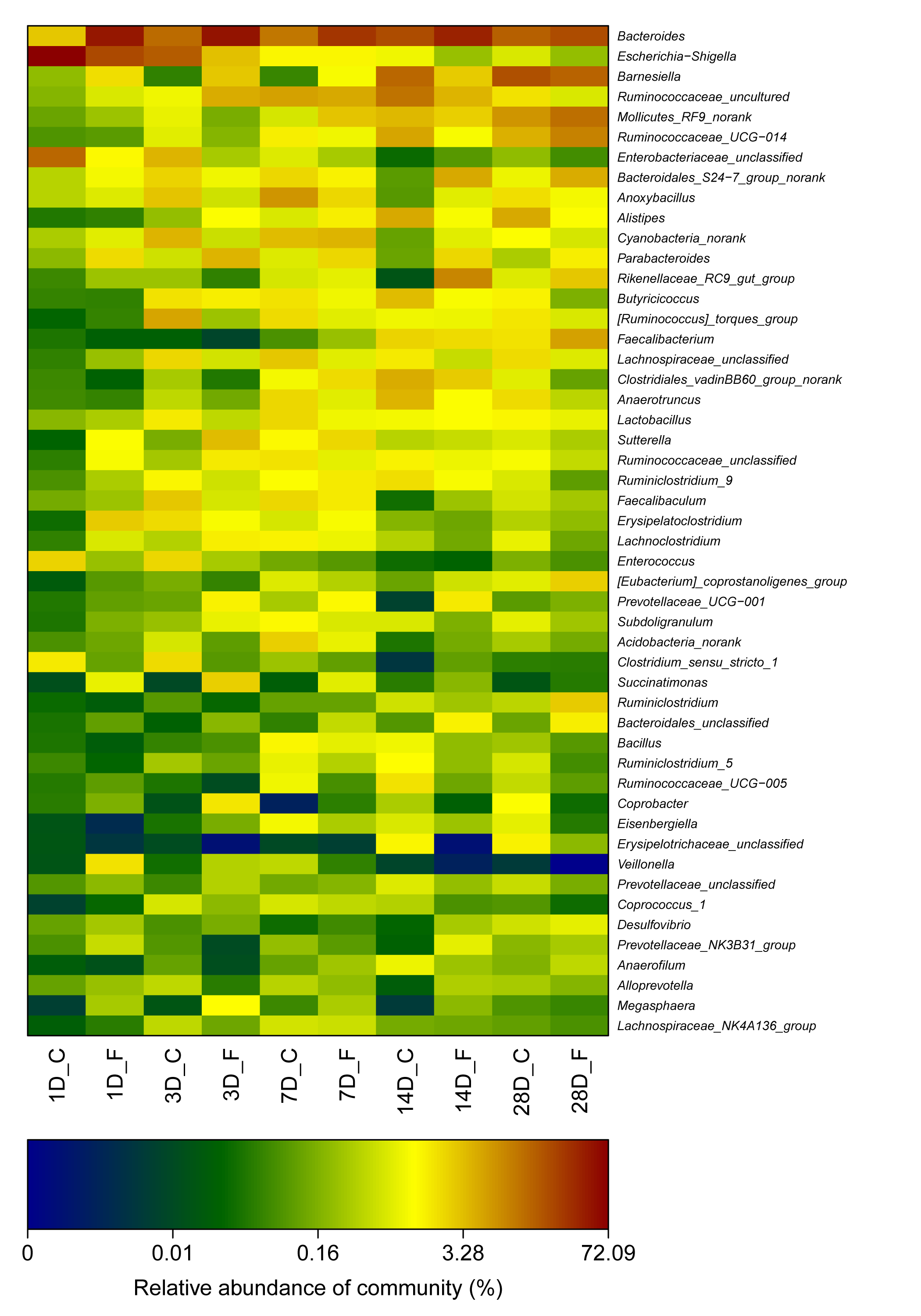
**Supplementary Figure S2.** The heatmap of microbial composition in the cecum of the broiler chickens on days 1, 3, 7, 14, and 28. *n* = 8 per group. C Group, control group; F group, fermentation broth group.
